# Prevalence of Bovine Rotavirus and Coronavirus in Neonatal Calves in Dairy Farms of Addis Ababa, Ethiopia: Preliminary Study

**DOI:** 10.1155/2021/5778455

**Published:** 2021-11-09

**Authors:** Motuma Debelo, Hayat Abdela, Asaminew Tesfaye, Abebaw Tiruneh, Gudina Mekonnen, Zerihun Asefa, Nebyou Moje

**Affiliations:** ^1^School of Veterinary Medicine, College of Agriculture and Veterinary Medicine, Jimma University, Jimma, Ethiopia; ^2^National Animal Health and Diagnostic Center, Sebeta, Ethiopia; ^3^School of Medical Laboratory Sciences, Institute of Health, Jimma University, Jimma, Ethiopia; ^4^School of Veterinary Medicine, Ambo University, Ambo, Ethiopia; ^5^College of Veterinary Medicine and Agriculture, Addis Ababa University, Bishoftu, Ethiopia; ^6^Faculty of Veterinary Medicine, Hawassa University, Hawassa, Ethiopia

## Abstract

**Background:**

Bovine rotavirus (BRV) and bovine coronavirus (BCoV) are the most common viral agents in neonatal calf diarrhea and result in serious economic consequences. The aim of the study was to determine the epidemiology of those viruses in randomly selected dairy farms of Addis Ababa.

**Methods:**

A cross-sectional study was conducted from November 2018 to April 2019 using a probability proportional to size (PPS) sampling technique. A total of 110 calves, less than 30 days of age, from 57 dairy herds were involved in the study. Associated factors of herds and calves were collected using semistructured interviews from farm owners and through physical observation of selected calves. Fecal samples were collected and analyzed using the sandwich ELISA method. Data generated from both semistructured interviews and laboratory investigation were analyzed using STATA_MP version 15.

**Results:**

From the total 110 calves, 42 (38.18%) had diarrhea during the survey. The prevalence of bovine rotavirus and coronavirus was 3.64% (4/110) and 0.91% (1/110), respectively. Diarrhea, feeding colostrum timing, and sex of the neonatal calves had statistically significant association with bovine rotavirus infection (*p* < 0.05). All rotavirus-positive neonatal calves were identified in small scale dairy farms and in dairy farms that reported mortality though they lack statistically significant association. Only one coronavirus case was detected among the neonatal calves. The case was identified among small scale herds and in a herd with diarrheal cases. The sex of the coronavirus calf was female, diarrheic, and among 11-20 days old.

**Conclusion:**

The prevalence of rotavirus and coronavirus infections in neonatal calves was seldom in dairy farms of the study area. Rotavirus was more common than coronavirus, and further studies should be initiated on other (infectious and noninfectious) causes of neonatal calf diarrhea in the area.

## 1. Introduction

Ethiopia basically constitutes an agrarian society wherein about 85% of the total population's socioeconomic activities are dependent on farming and animal husbandry. Livestock plays an important role for the majority of the Ethiopian population [1]. Dairy production is one of the important sources of food and income generation in Ethiopian livestock-based society. Dairy farms are concentrated in urban and periurban areas of the country due to better availability of milk market in the area. The substantial demand-supply variance in milk and milk products for the major urban centers in Ethiopia is a great opportunity for the development and flourishing of periurban dairy farms [2, 3].

The success of any breeding program as well as the future of dairy farms depends upon the rate of survival of the calves produced. Accordingly, calf morbidity and mortality are of great concern of dairy farms because most of the dairy farms are confronted with acute problems of calf morbidity and mortality [4]. Diarrhea in neonatal period and pneumonia in older calves are known to be responsible for most of the calfhood morbidity and mortality [5, 6]. Neonatal calf diarrhea is a complex of different disease syndromes that causes economic losses directly through mortality and cost of treatment and indirectly from poor growth performance [7].

Calf diarrhea is related with environmental, management-related, nutritional, and physiological factors either alone or in synergy with different infectious agents such as protozoans, bacteria, and/or viruses. Among these infectious agents that have been implicated in calf diarrhea, viruses: bovine coronavirus (BCoV), bovine rotavirus (BRV), and bovine viral diarrhea virus (BVDV); bacteria: *Salmonella* species, *E. coli* K99^+^, and *Clostridium* species; and protozoa: *Cryptosporidium* species are some examples that lead to major economic losses in dairy farms [5, 8]. Among the viral agents, BRV and BCoV are the most commonly associated causes of neonatal diarrhea and economically important pathogens [9].

Identification of possible causative agents during outbreaks of diarrhea is important to allow targeted preventative measures, such as vaccination and determination of possible risk factors or sources of infection [10]. In Ethiopia, calf diarrheal diseases caused by bacteria and protozoan parasites get the main concern, and viral causes are overlooked. Therefore, the aim of this study was to determine the prevalence of the two viral causes of diarrhea, BCoV and BRV, among neonatal calves in dairy farms of Addis Ababa, Ethiopia.

## 2. Methods

### 2.1. Study Area

**Figure 1:**
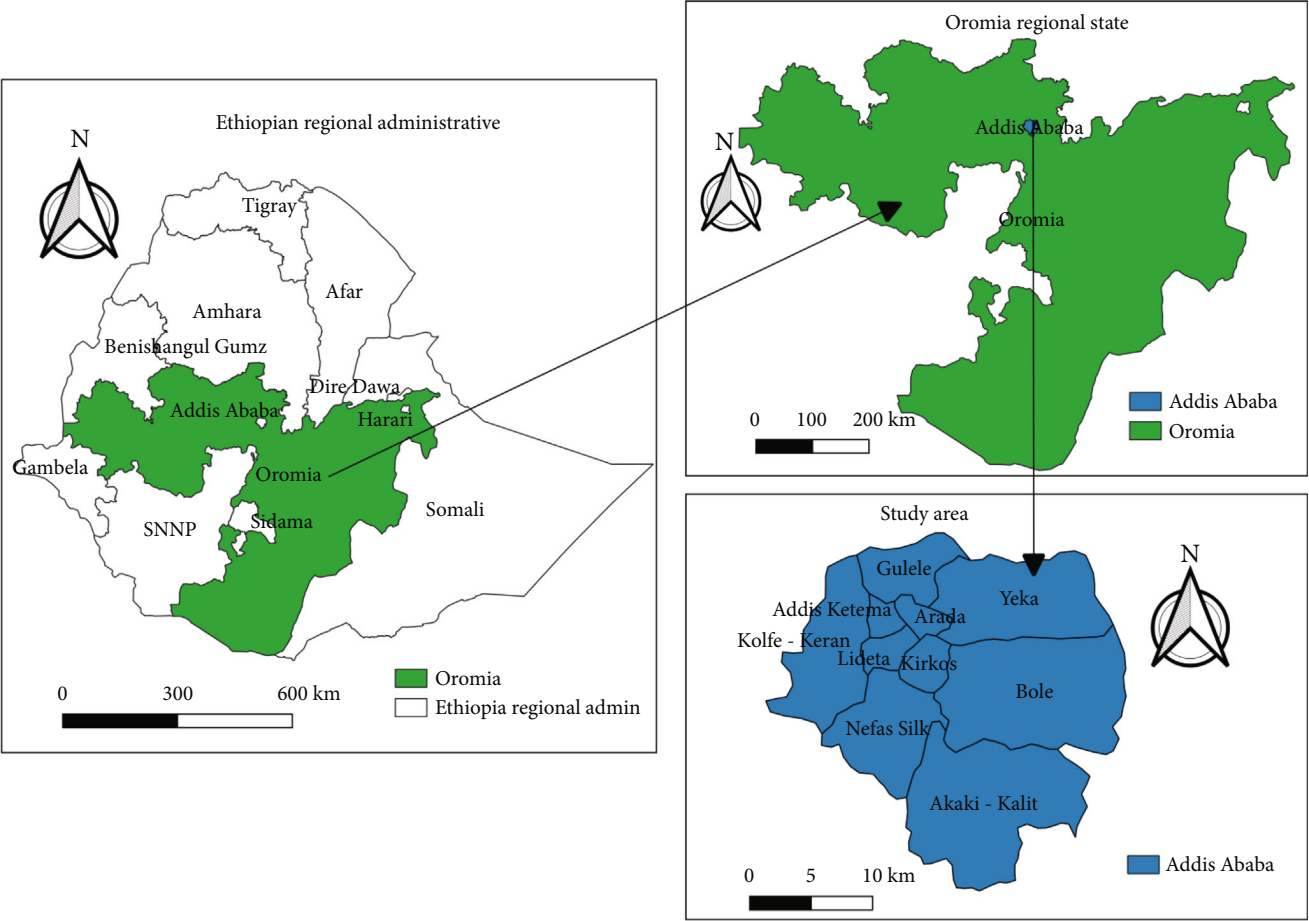
Map of the study area, Addis Ababa, Ethiopia.

Addis Ababa is located at a latitude and longitude of 9^o^1′48^″^N and 38°44′24^″^E, respectively, and at an average altitude of 2500 m above sea level. It receives an average annual rainfall of 1800 mL with average temperature of 21°C. The relative humidity varies from 70 to 80% during the rainy season and from 40 to 50% during the dry season. The city has 10 subcities and 117 woredas (Figure 1). The economic activities in Addis Ababa are diverse; according to official statistics from the federal government, 16,602 people are engaged in agriculture. In addition to the residents of rural parts of Addis Ababa, the city dwellers also participate in animal husbandry and cultivation of gardens. Addis Ababa and its periurban areas have 62,166 bovine, 22,647 ovine, 7,531 equine, 5,597 caprine, and 330,000 avian species in 2008/2009 [11].

### 2.2. Study Animals

All dairy farms in ten subcities of Addis Ababa were contacted, and 57 dairy farms/herds agreed to participate in the study.

The study animals were neonatal calves (up to 30 days of age) with and without diarrhea during the survey. Neonatal calves were pure Holstein Friesian (Bos primigenius) and crossbred of Zebu breed and Bos primigenius kept under semi-intensive or intensive management system.

### 2.3. Study Design, Sample Size, Sample Collection, and Examination

A cross-sectional study was conducted from November 2018 to April 2019 among randomly selected 110 neonatal calves in 57 dairy herds. The sample size was calculated using a single population proportion formula (X = Z*α*/22∗*p*∗(1 − *p*)/MOE^2^), considering *p* = 7.2 [12], *d* = 0.05, and 10% none response rate (103 + 10 = 113). The calves were selected from 57 dairy herds using a probability proportional to size (PPS) sampling technique, and dairy farms were visited once.

The research was ethically reviewed and approved by the Ethics Committee of Jimma University College of Agriculture and Veterinary Medicine. Consent of the owners was obtained to collect fecal samples from their calves and for the interview.

Questionnaire data was collected using semistructured questionnaire from each farm owners including age, colostrum feeding and timing, amount, and management types. Name of the farm, sex, type of breed, herd size, calf mortality in the herd, presence of diarrhea, and consistency of diarrhea were also recorded for each calf on proper recording format. Diarrhea was considered if feces are semiliquid to liquid, with or without other abnormal characteristics such as presence of blood. Any calf without these characteristics of feces was considered nondiarrheic or healthy. Diarrhea was distinguished by the veterinarian during the survey. Presence of calf mortality in the herd was the mortality of neonatal calf in that specific herd in the last 12 months.

Dairy farms were stratified in to small (≤19 dairy animals), medium (20-49 dairy animals), and large (≥50 dairy animals) scale dairy farms.

Fecal samples of approximately 32 grams were collected in a sterile tube labeled with animal ID after cleaning of the anal area with a paper towel from both diarrheic and nondiarrheic calves using disposable latex gloves. Samples were placed into sterile universal bottle and labeled then transported to National Animal Health and Diagnostic Center located in Sebeta, Ethiopia, using an ice box containing ice packs. The samples were stored at +4°C until time of processing.

### 2.4. Laboratory Analysis

Fecal samples were allowed to thaw at room temperature and diluted volume by volume into a dilution buffer until it allows pipetting of fecal suspensions. Multiscreen Ag ELISA Calf digestive (BIO K 314/1, Belgium) was used to detect BCoV and BRV antigens in the fecal suspensions. The sandwich ELISA procedure was performed according to the manufacturer instructions as detailed in the kit (kit reference BIO K 314/1). Any sample that yields optical density (OD) of ≥600% (6) for rotavirus and ≥700% (7) for coronavirus was considered as positive. The test is validated only if the positive control antigens for coronavirus and rotavirus yield OD of >1000% (10) at 10 minutes. All fecal samples were tested once, and positive samples were repeated.

### 2.5. Data Analysis

Data generated from questionnaire and laboratory investigation was entered, coded into Microsoft Excel 2007 spreadsheet, and analyzed using STATA version 15. *Χ*^2^ value was calculated using descriptive statistics model.

All statistical tests were considered significant if the *p* value is <0.05.

## 3. Results

### 3.1. Overall Prevalence

The prevalence of BRV and BCoV was 3.64% (95CI: 0.99-9.04) and 0.91% (95% CI: 0.0002-4.96), respectively. The overall prevalence of BRV and BCoV was 4.55% (95% CI: 1.49-10.29) (Table 1).

Only one coronavirus-positive calf was detected among the neonatal calves. The case was identified in a small scale herd and in a herd with diarrheal cases. The coronavirus-positive calf was female, diarrheic, and among age groups of 11-20 days old.

### 3.2. Prevalence of Bovine Rotavirus and Herd- and Individual-Level Risk Factors

A total of four (3.64%) rotavirus-positive calves were detected. All rotavirus-positive cases were identified in small scale herd size, and one of the herds had two rotavirus-positive calves. There was no BRV-positive calves in the medium and large scale herd sizes. However, the herd size lacks statistically significant association with BRV infection. Presence of diarrhea among herd (*p* = 0.009) and colostrum feeding time (*p* = 0.033) had statistically significant association with prevalence of rotavirus (Table 2). Other herd-level variables including history of mortality, ways of colostrum feeding, bedding, colostrum amount, and calf pens had no statistically significant association with the infection of rotavirus.

From the total 110 calves, 42 (38.18%) had diarrhea during the survey. Presence of diarrhea in neonatal calves (*p* = 0.01) and watery diarrhea type (*p* = 0.01) had statistically significant association. All BRV-positive calves were male, and sex of calves had statistically significant association with rotavirus infections (*p* = 0.04) (Table 3). The age group of calves lacks statistically significant association with rotavirus infection.

## 4. Discussion

The overall prevalence of BRV and BCoV in the study area was 4.55%. The result of the present study showed that BRV had higher prevalence, 3.64% (4/110), than BCoV, 0.9%(1/110), in the study area. A study conducted in Central Oromia, Ethiopia, documented higher prevalence of rotavirus than coronavirus [12]. Other studies conducted elsewhere in the world corroborate with our findings [13, 14]. However, a study conducted in 1992 by Abraham et al. in Ethiopia reported higher prevalence of coronavirus and rotavirus, 38.9% and 16.7%, respectively [15]. The inconsistency of the result might be from year of study, environmental factors, farm management practices exercised, hygienic measures, and geographical locations. Moreover, we recommend further study with advanced sample size.

In the current study, the association of BRV and BCoV infection was measured with herd- and individual-level risk factors. Male calves had higher possibility of rotavirus acquisition as compared to their counterpart females. The finding was in agreement with the reports of a study conducted in North Dakota and Himachal Pradesh [16]. This can be explained as size of male at birth is assumed to induce dystocia and consequently decrease colostrum absorption. Furthermore, more care is given to female calves than males because of their economic importance and replacement stock strategy [17]. However, a study conducted in Algeria revealed higher prevalence of rotavirus among female calves than males [18].

The prevalence of both BRV and BCoV was high in calves at the age group of 11-20 days with 3 (7.9%) and 1 (2.6%) occurrences, respectively. This may be due to the lack of natural immunity against the two infections and a decrease in passive immunity [19]. In calves 1-10 days of age, only infection by rotavirus was detected (1 (3.2%)). This may be related to the attention and care given to neonatal calves at this age range in the study area. None of the pathogens were detected in the third age group (21-30 days). These may be justified as increased natural immunity against the pathogens as calves reach beyond 3 weeks old [20].

Diarrhea is among the major causes of neonatal calf morbidity and mortality. In this study, both viral agents (9.5% for BRV and 2.4% for BCoV) were highly prevalent among diarrheic calves. Although BRV infections had statistically significant association with diarrheic calves, many of the BRV-negative calves also had diarrhea. This might be due to numerous potential etiologic agents of diarrheal diseases in neonatal calves including other viral, bacterial, and protozoan pathogens [20]. None of the nondiarrheic calves showed infection by both pathogens in the present study. Studies conducted elsewhere also reported the absence of rotavirus and coronavirus in nondiarrheic calves [21, 22]. Moreover, the consistency of diarrhea for the positive samples were watery type for all rotavirus cases and smooth for the coronavirus case which might be due to the effect of the viruses mainly on the small intestine villi [23, 24].

The prevalence of rotavirus was significantly associated with colostrum feeding time of the calves. Additionally, the way of colostrum feeding and its amount were also associated with the occurrence of the disease. The prevalence of BCoV (2%) and BRV (4.1%) was high in herds that feed colostrum with bucket than suckling. This may be due to contamination of buckets with calf feces that also contaminate colostrum while feeding calves. Calves getting colostrum after 2 hrs of birth had also shown a high prevalence rate of rotavirus (22.2%) and coronavirus (5.3%) as compared to <1 hr in the herd. Each hour of delay in colostrum ingestion in the first 12 hours of age increases the chance of calves becoming ill. Delaying colostrum intake is known to decrease intestinal immunoglobulin and fat-soluble vitamin absorption. This is mainly related to the absence of immunoglobulin absorption into the circulation after 24 hours due to closure of the calf's gut [25]. The time between birth and the first feeding is the prime factor for the failure of passive transfer of colostral immunity. Another study conducted in Ethiopia also reported that calf mortality is significantly higher in those that got colostrum late after birth [26]. A higher infection rate of BRV (8.3%) and BCoV (4.2%) was observed in farms that gave colostrum below 1 liter. This can be justified as the low amount of colostrum provision to the calves resulted in high BRV and BCoV infection. Failure of passive transfer will increase susceptibility of calves for bacterial and viral infections. Therefore, adequate amount of IgG mass (>100 grams IgG) can be achieved if the calf received 4 liters of colostrum or a minimum of 3 liters [27]. Moreover, all cases of BRV were detected from small scale dairy farms though it lacks statistically significant association. The possible cause of increased BRV cases in small scale dairy farms might be poor management observed during the survey.

### 4.1. Limitation of the Study

The number of neonatal calves was small, and molecular techniques was not used for better detection of BRV and BCoV.

## 5. Conclusion

Data from this study showed that both BRV and BCoV infections were involved in neonatal calf diarrhea. Therefore, it is advisable that awareness should be created to dairy farm owners and attendants on overall farm management specifically on proper cow-calf management, feeding, and hygienic practices in dairy farms. Further studies on other (infectious and noninfectious) causes of calf diarrhea would be carried out and proper vaccination program could be designed for protection of calf diarrhea.

## Figures and Tables

**Table 1:** Overall prevalence of rotavirus and coronavirus in neonatal calves, Addis Ababa.

Viruses	No. positive (*N* = 110)	Prevalence (95% CI)
Rotavirus	4	3.64 (0.99-9.04)
Coronavirus	1	0.91 (0.0002-4.96)
Total	**5**	4.55 (1.49-10.29)

**Table 2:** Association of herd risk factors with rotavirus prevalence (*n* = 57).

Risk factors	No. of herds examined	No. of positive herds	Prevalence (95% CI)	*χ* ^2^	*p* value
Herd size					
Small scale	34	3	8.8 (1.85, 23.67)	2.14	0.343
Medium scale	18	0	0.0 (0.0, 0.0)		
Large scale	5	0	0.0 (0.0, 0.0)		
Diarrhea					
Yes	18	3	16.7 (3.58, 41.42)	6.86	0.009^∗^
No	39	0	0.0 (0.0, 0.0)		
Mortality					
Yes	28	3	10.7 (2.23, 28.22)	3.28	0.07
No	29	0	0.0 (0.0, 0.0)		
Colostrum feeding					
Bucket	49	2	4.1 (0.50, 13.98)	0.98	0.323
Suckling	8	1	12.5 (0.31, 52.65)		
Colostrum timing					
<1 hr	29	0	0.0 (0.0, 0.0)	6.8	0.033^∗^
1-2 hr	19	1	5.3 (0.13, 26.03)		
>2 hr	9	2	22.2 (2.81, 60.00)		
Bedding					
Yes	40	3	7.5 (1.57, 20.38)	1.35	0.246
No	17	0	0.0 (0.0, 0.0)		
Colostrum amount					
<1 L	24	2	8.3 (1.03, 27.00)	0.89	0.64
1-2 L	28	1	3.6 (0.0, 18.35)		
>2 L	5	0	0.0 (0.0, 0.0)		
Calf pen					
Individual	25	1	4 (0.10, 20.35)	0.14	0.71
Group	32	2	6.2 (0.76, 20.81)		

^∗^Significant at *p* < 0.05.

**Table 3:** Association of individual risk factors with rotavirus prevalence.

Risk factors	No. of calves sampled	No. of positive calves	Prevalence (95% CI)	*χ* ^2^	*p* value
Sex					
Male	55	4	7.3 (2.01, 17.58)	4.15	0.04^∗^
Female	55	0	0.0 (0.0, 0.0)		
Diarrhea					
Diarrheic	42	4	9.5 (2.60, 22.62)	6.72	0.01^∗^
Nondiarrheic	68	0	0.0 (0.0, 0.0)		
Age					
1-10 days	31	1	3.2 (0.0, 16.70)	0.47	0.79
11-20 days	39	3	7.9 (7.77, 7.99)		
21-30 days	40	0	0.0 (0.0, 0.0)		
Consistency of diarrhea					
Normal	87	0	0.0 (0.0, 0.0)		
Smooth and mucoid	5	0	0.0 (0.0, 0.0)		
Watery	13	4	30.8 (9.10, 61.42)	30.97	0.01^∗^
Bloody	5	0	0.0 (0.0, 0.0)		

^∗^Significant at *p* < 0.05.

## Data Availability

The data used to support the findings of this study are included within the article.

## Abbreviations

BCoV: Bovine coronavirus
BRV: Bovine rotavirus
OD: Optical density.
